# Supplementary material for: Experiences of adult children caring for parents aging in place
Source: PLoS One. 2025 Sep 16;20(9):e0332336. doi: 10.1371/journal.pone.0332336 (PMC12440213; doi:10.1371/journal.pone.0332336)
Supplement: Attachment — Submitted filename: comments.docx [file pone.0332336.s001.docx]

Through 22 semi-structured interviews and using Strauss and Corbin’s coding method, the authors developed a novel Challenge-Acceptance Theory (CAT) that explains how caregivers transition from challenge to acceptance. The study presents valuable insights for gerontology, family studies, and health care professionals.

**Comments:**

The manuscript would benefit from:

A more concise model figure with clearly labeled components.

Clarifying whether this theory is new, how it builds upon or diverges from similar theories (e.g., stress-coping model, caregiver burden model).

Discussing how CAT could be applied practically (e.g., in interventions, assessments, caregiver training).

Consider discussing how the researcher’s background, potential bias, or relationship with participants may have influenced interpretation.

Were bracketing or peer debriefings used to reduce bias?

Discussion:

Compare more explicitly how this study adds new knowledge to existing literature.

Avoid re-stating results in long form—focus on synthesis and implications.

Clarify if any gender-based differences were observed in caregiving experiences (this is implied but not fully explored).
