# Supplementary material for: Experiences of adult children caring for parents aging in place
Source: PLoS One. 2025 Sep 16;20(9):e0332336. doi: 10.1371/journal.pone.0332336 (PMC12440213; doi:10.1371/journal.pone.0332336)
Supplement: Attachment — Submitted filename: Response to Reviewers.docx [file pone.0332336.s003.docx]

Response to reviewers’ comments

***Reviewer #1:***

1. **This study is well-perceived and executed with a proper methodology. All sections are described properly. However, concluding research on a relatively small sample with a theory is too early to draw a conclusion.**

Thank you for your thoughtful comments and positive evaluation of the methodology and structure of our manuscript. We understand the concern regarding the development of a theory based on a relatively small sample size.

However, grounded theory methodology does not prioritize large sample sizes. Rather, its focus is on generating a theory that is grounded in data through systematic collection and analysis until theoretical saturation is reached. According to (2024) and Corbin and Strauss (2015), smaller samples are appropriate in grounded theory research because they allow for in-depth, iterative engagement with the data, which is necessary for meaningful theory development. In our study, we conducted 22 semi-structured interviews and reached theoretical saturation, where no new categories or concepts were emerging. We have also included a section on the strengths and limitations to acknowledge that further research is necessary to refine and validate this theory in broader and diverse caregiving populations (pg. 29).

We hope these clarifications will address the reviewer’s concern while preserving the integrity of our grounded theory approach.

2. **It’s true that without proper training, caring for elderly people is difficult, but it should also be in view that the given example of the study include people over 45 taking care of elderly people. moreover, this study is specified to middle-aged people taking care of elderly. What about young people taking care of the elderly? Different age groups were also to be included to draw a theory as a conclusion.**

We thank the reviewer for this insightful observation. Indeed, caregiving experiences can differ across age groups, and younger caregivers may encounter distinct challenges and perspectives when compared to middle-aged caregivers.

The scope of our study was intentionally focused on adult children caregivers who had provided care for at least one year in a home setting. While we did not exclude younger adults by design, the final sample, recruited using snowball sampling, included 41% of adults aged 29-55 years old. Our sample reflects the demographic reality in many caregiving contexts where middle-aged and younger adult children are often the primary caregivers of aging parents.

We politely disagree with the comment that our study is specific to middle-aged people taking care of the elderly. We have a sizable number (41%) of people aged 29-55 taking care of the elderly in our study.

***Reviewer #2:***

Through 22 semi-structured interviews and using Strauss and Corbin’s coding method, the authors developed a novel Challenge-Acceptance Theory (CAT) that explains how caregivers transition from challenge to acceptance. The study presents valuable insights for gerontology, family studies, and health care professionals.

***Comments:***

3. **The manuscript would benefit from: A more concise model figure with clearly labeled components. Clarifying whether this theory is new, how it builds upon or diverges from similar theories (e.g., stress-coping model, caregiver burden model).**

We thank the reviewer for these thoughtful and constructive suggestions. In response:

***Revised Model Figure:***

We have updated Figure 1 to present a more concise and clear representation of the Challenge-Acceptance Theory (CAT) - (see revised model). The revised model includes clearly labeled components reflecting the emergent themes and their interconnections based on grounded theory coding. This visual clarity enhances readers’ understanding of the theory’s structure.

***Clarification of Theoretical Contribution:***

We have added a paragraph to clarify that CAT is a novel, empirically derived theory (pg. 26). While it intersects with existing frameworks such as the stress-coping model (Lazarus & Folkman, 1984) and the caregiver burden model (Zarit et al., 1980), CAT distinguishes itself by emphasizing the processual and transformative nature of caregiving. Rather than viewing caregiving solely as a burden or stressor, CAT illuminates the journey through challenge to acceptance through the caregiving process. .

***Practical Applications:***

In response to the reviewer’s suggestion, we have also included a section that discusses the potential applications of CAT in real-world settings (pgs. 26-27). Specifically, CAT may inform the development of targeted interventions aimed at caregiver support, training programs that foster adaptive coping strategies, and assessment tools to identify caregivers at different phases of the challenge-acceptance continuum. Healthcare professionals and social service agencies can use the CAT framework to tailor services based on where caregivers are situated in this dynamic process..

We believe these revisions strengthen the theoretical and practical contributions of our manuscript and appreciate the reviewer’s input.

4. ***Consider discussing how the researcher’s background, potential bias, or relationship with participants may have influenced interpretation. Were bracketing or peer debriefings used to reduce bias?***

We appreciate the reviewer’s important observation regarding the role of researcher reflexivity in qualitative research. In response, we have added a subsection in the Methods section under a new subheading titled “Researcher Reflexivity and Rigor” to describe how we addressed potential biases throughout the research process (pg. 8).

Specifically, the primary researcher engaged in ongoing reflexive journaling to monitor personal assumptions and preconceptions. In addition, bracketing techniques were employed to set aside prior experiences related to caregiving, and peer debriefing sessions were conducted regularly with a qualitative research consultant. These strategies helped enhance the credibility and confirmability of the findings by allowing for critical examination of emerging themes and coding decisions. We believe these methodological safeguards strengthened the rigor and trustworthiness of the theory development process.

5. **Discussion: Compare more explicitly how this study adds new knowledge to existing literature. Avoid re-stating results in long form—focus on synthesis and implications. Clarify if any gender-based differences were observed in caregiving experiences (this is implied but not fully explored).**

We appreciate the reviewer’s feedback regarding the need for clearer articulation of the study’s contribution to the literature. In response, we have added a paragraph under the CAT model to highlight how the Challenge-Acceptance Theory (CAT) extends existing knowledge by introducing a process-oriented, meaning-centered framework that captures the emotional and cognitive transitions adult children undergo while caring for aging parents (pg. 26).

While much of the existing literature centers on caregiver burden, role strain, or stress-coping mechanisms, our theory uniquely synthesizes both the hardships and adaptive responses into a sequential framework grounded in the experience. The study contributes novel insights by framing caregiving not as a static burden, but as a dynamic trajectory toward meaning-making and acceptance.

Although the sample size limited detailed subgroup analysis, our findings revealed no significant gender differences in the identified themes. Nevertheless, we recommend that future studies explore caregiving experiences based on gender-specific roles (pg. 30).

***Reviewer #3:*** -

6. **A Data Availability Statement would be formally included to improve openness. Briefly stating that while anonymised extracts of the interview are available and full interview data is secret, additional access may be granted upon reasonable request. A suggested text that you can modify as necessary: "The paper contains the data corroborating the study's findings. Complete interview transcripts are not publicly accessible due to ethical and confidentiality constraints, however, they might be provided upon the relevant author's reasonable request, subject to institutional consent.- Even if it was done informally, think about quickly outlining how you made sure that there was variety in terms of gender and race/ethnicity during snowball sampling (for example, by using targeted outreach or particular recruitment objectives). This will highlight your sampling strategy's resilience even more.-**

We thank the reviewer for this helpful suggestion regarding data transparency and sampling strategy. In response:

***Data Availability Statement:***

We have added a formal Data Availability Statement to comply with PLOS ONE’s openness guidelines (pg 7). “While full transcripts cannot be publicly shared due to confidentiality agreements with participants, anonymized excerpts that support the findings are included in the manuscript. We note that additional data may be made available upon reasonable request, subject to institutional approval.”

***Sampling Diversity and Strategy:***

Although our snowball sampling method was informal, we intentionally sought demographic diversity among participants. Recruitment was initiated through multiple community organizations, including churches and senior centers, serving diverse populations (see pg. 6). This informal stratification approach contributed to variation in our sample and enhanced the richness and applicability of the grounded theory generated.

7. **Kindly indicate which version was utilized (e.g., NVivo 12, NVivo 20).-**

We appreciate the reviewer’s request for clarity regarding the version of NVivo software used in the data analysis. We have updated the Methods section to indicate that NVivo 12 Plus (pg. 9) was used for thematic analysis and coding.

8. **As some publications now require it, think about emphasizing the committee name and approval protocol number in the Methods section. –**

Thank you for the reminder regarding ethical compliance reporting. We rechecked the manuscript, and the requested information was on page 6 of the manuscript. Thank you.

9. **I suggest adding data about the benefits of physical therapy for improving the health of senior citizens to your discussion of falls and physical exercise. Your discussion of caregiving techniques may be enhanced by research showing the value of mobility and balance training in preventing falls. Studies [DOI: doi.org/10.47363/JPMRS/2023(5)192] that describe how physical therapy therapies might lower fall risks and enhance general health may be of use to you. They offer a useful framework for caregivers of elderly parents who have mobility issues.**

We thank the reviewer for this valuable suggestion and acknowledge recent findings, such as Singh et al. (2023), which emphasize the benefits of physical therapy in improving mobility, balance, and fall prevention among older adults. However, our study did not focus on falls, nor did participants identify falls as a caregiving challenge or acceptance factor. Including this topic in the discussion would diverge from the central aims of our study.

**References:**

Charmaz, K. (2024). Constructing grounded theory (3rd ed.). SAGE Publications.

Corbin, J., & Strauss, A. (2015). Basics of qualitative research: Techniques and procedures for developing grounded theory (4th ed.). SAGE Publications.

Lazarus, R. S., & Folkman, S. (1984). Stress, appraisal, and coping. Springer Publishing.

Zarit, S. H., Reever, K. E., & Bach-Peterson, J. (1980). Relatives of the impaired elderly: Correlates of feelings of burden. The Gerontologist, 20(6), 649–655. <https://doi.org/10.1093/geront/20.6.649>

Singh, S., Mehra, R., Kaur, H., & Verma, P. (2023). Role of physiotherapy in the prevention of falls among elderly individuals: A systematic review. *Journal of Physical Medicine, Rehabilitation & Sports*, 5(5), 192. <https://doi.org/10.47363/JPMRS/2023(5)192>
